# Targeted Next-Generation Sequencing Identification of Mutations in Disease Resistance Gene Analogs (RGAs) in Wild and Cultivated Beets

**DOI:** 10.3390/genes8100264

**Published:** 2017-10-11

**Authors:** Piergiorgio Stevanato, Chiara Broccanello, Luca Pajola, Filippo Biscarini, Chris Richards, Lee Panella, Mahdi Hassani, Elide Formentin, Claudia Chiodi, Giuseppe Concheri, Bahram Heidari

**Affiliations:** 1 Department of Agronomy, Animals, Natural Resources and Environment-DAFNAE, University of Padova, 35020 Legnaro (Padova), Italy; stevanato@unipd.it (P.S.); chiarabr87@yahoo.it (C.B.); lucapajola@yahoo.it (L.P.); claudia.chiodi91@gmail.com (C.C.); giuseppe.concheri@unipd.it (G.C.); 2 Cardiff University, School of Medicine, Heath Park, CF14 4XN Cardiff, UK; 3 Consiglio Nazionale delle Ricerche (CNR), 20133 Milan, Italy; filippo.biscarini@ibba.cnr.it; 4 USDA-ARS, National Laboratory for Genetic Resources Preservation, Fort Collins, 80521 CO, USA; Chris.Richards@ars.usda.gov; 5 Colorado State University, Department of Soil and Crop Sciences, Fort Collins, 80521 CO, USA; lee.panella@colostate.edu; 6 Department of Crop Production and Plant Breeding, School of Agriculture, Shiraz University, 7144165186 Shiraz, Iran; bheidari@shirazu.ac.ir (B.H.); mehdi_hasani@yahoo.com (M.H.); 7 Sugar Beet Seed Institute (SBSI), 315854114 Karaj, Iran; 8 Department of Biology, University of Padova, 35131 Padova, Italy

**Keywords:** AmpliSeq, Ion Torrent sequencing, mutation discovery, single nucleotide polymorphism, sugar beet, disease resistance, rhizomania and nematodes, gene *Bv8_184910_pkon*

## Abstract

Resistance gene analogs (RGAs) were searched bioinformatically in the sugar beet (*Beta vulgaris* L.) genome as potential candidates for improving resistance against different diseases. In the present study, Ion Torrent sequencing technology was used to identify mutations in 21 RGAs. The DNA samples of ninety-six individuals from six sea beets (*Beta vulgaris* L. subsp. *maritima*) and six sugar beet pollinators (eight individuals each) were used for the discovery of single-nucleotide polymorphisms (SNPs). Target amplicons of about 200 bp in length were designed with the Ion AmpliSeq Designer system in order to cover the DNA sequences of the RGAs. The number of SNPs ranged from 0 in four individuals to 278 in the pollinator R740 (which is resistant to rhizomania infection). Among different groups of beets, cytoplasmic male sterile lines had the highest number of SNPs (132) whereas the lowest number of SNPs belonged to O-types (95). The principal coordinates analysis (PCoA) showed that the polymorphisms inside the gene *Bv8_184910_pkon* (including the CCCTCC sequence) can effectively differentiate wild from cultivated beets, pointing at a possible mutation associated to rhizomania resistance that originated directly from cultivated beets. This is unlike other resistance sources that are introgressed from wild beets. This gene belongs to the receptor-like kinase (RLK) class of RGAs, and is associated to a hypothetical protein. In conclusion, this first report of using Ion Torrent sequencing technology in beet germplasm suggests that the identified sequence CCCTCC can be used in marker-assisted programs to differentiate wild from domestic beets and to identify other unknown disease resistance genes in beet.

## 1. Introduction

Sugar beet is an important crop that supplies around 20% of the sugar consumed worldwide. It is cultivated in over 50 countries [1]. The crop is damaged by biotic and abiotic stresses and the development of new varieties that are tolerant under adverse conditions is one of the main breeding challenges [2]. Rhizomania and nematode-infections are the most widespread diseases, respectively induced by Beet Necrotic Yellow Vein Virus [3] and beet-cyst nematode (*Heterodera schachtii* Schm.) [4]. The only efficient and cost-effective strategy to control these diseases is to introgress resistance genes into commercial sugar beet varieties. Today, the market share of varieties encompassing better disease resistances is increasing greatly. In particular, the market-share of double-resistant (rhizomania and nematodes) varieties is projected to increase by up to 70% by 2021 [1,3].

The introgression of resistance genes from wild relatives to sugar beet cultivars remains fundamentally important. The sea beet (*Beta vulgaris* L. ssp. *maritima* (L.) *Arcang.*), the wild progenitor of the domesticated sugar beet, provides useful sources of resistance to many diseases [5]. In the absence of resistance, the cultivation of sugar beet would be practically impossible in most areas that are cultivated at the present moment. Wild relatives of domesticated sugar beet are therefore widely used in breeding programs to improve pest and disease resistance. The first source of a resistance gene for rhizomania (*Rz1*) was identified in the WB258 sea beet population from the Adriatic coast of Italy [6]. Resistance gene analogs (RGAs) are a large family of genes that share conserved domains and structural features. As such, RGAs can be identified from sequenced genomes using bioinformatics approaches [7]. Despite the fact that thousands of RGAs have been identified through sequence homology, only a handful have been cloned and fully characterized. The latter offer information about the structure, function and evolution of resistance genes, and have delivered cultivars with novel resistance [8,9,10,11]. *Hs1^pro-1^* is the only resistance gene cloned from sugar beet [12]. This gene delivers resistance to infection by the beet nematode. 

Single nucleotide polymorphisms (SNPs) can be used to analyze variations inside the sequence of genes with typical RGA features. SNPs present advantages compared to other genetic marker types. SNPs are abundant, stable, co-dominant, amenable to an automated method of detection, accurate, and relatively inexpensive. Sugar beet has an estimated genome size of about 730 Mbp, and a 567 Mbp reference sequence with 26,923 annotated protein-coding genes has recently been published [13]. The recent release of the sugar beet reference genome has facilitated the discovery and mapping of SNPs in association with different traits.

The Sanger sequencing method, introduced in 1977, allowed the identification of the sequence of genes and frequency of mutations inside genes [14]. However, this method is laborious and time-consuming and requires large amounts of DNA, with a relatively low sensitivity and throughput [15]. Allele-specific PCR-based techniques, TaqMan-based methods, and high melting resolution (HRM) analysis have revolutionized high-throughput genotyping approaches [16]. The limitations of these techniques are that only small regions of the targeted genes are screened and not all the associated variants can be identified efficiently. Alternatively, the rapid developments in next-generation sequencing (NGS) technologies allow simultaneous sequencing of multiple genes in a single cost-effective assay [17]. NGS is used to identify specific changes in DNA by rapidly and simultaneously sequencing multiple gene targets within multiple samples. Capistrano-Gossman et al. [18] used SNP markers in an open-pollinated wild beet (*B. maritima*) population to demonstrate the potential of wild germplasms for sugar beet improvement. The results of the same study indicated that access to the DNA sequence of resistance gene *Rz2* opens the path to improve rhizomonia resistance. Grimmer et al. [19] highlighted the potential for sugar beet breeders to exploit the diverse wild beet germplasm for the introgression of genes in order to make commercial varieties resistant to viral infections.

Molecular marker technologies greatly facilitate introgression of disease resistance traits into sugar beet breeding programs. Ion Torrent Ampliseq designer is a new technology with the ability to resequence genes and search for associations based on the Disease Research Area database [20]. It has superior quality, specificity and coverage, which supports short (140–175 bp), medium (275 bp) and long (375 bp) sized PCR amplicons [21]. This technology is currently used in genetic screening, and as a diagnostic method for the identification of mutations inside genes. In the present study, Ion Torrent sequencing technology with the personal genome machine (PGM) was used to detect genetic mutations inside 21 selected RGAs for rhizomania and nematode infections in wild and cultivated beets. This is the first report of using this accurate and efficient technology in sugar beet research. Domestication and continuous selection for desirable traits made cultivated beets susceptible to many diseases as compared to the wild beet germplasms. Comparison of wild and domesticated beets could assist the identification of sources of disease resistance and mutations under selective pressures. The diagnostic gene-specific SNP markers that have been identified are likely to be useful in screening beet germplasms for resistance genes which may be used in marker-assisted selection (MAS) breeding programs for sugar beet.

## 2. Materials and Methods

### 2.1. Plant Material

Ninety-six individual samples of beet were chosen in the present study. Samples were derived from 5 beet groups comprising cytoplasmic male sterile lines, hybrid varieties, O-types, pollinators, and wild-type accessions (*B. maritima*). The seeds from this germplasm were produced in 2012 at the University of Padova, Italy. The seeds were germinated in soil pots with daily watering. Ten days after planting, seedlings were collected for DNA isolation.

### 2.2. DNA Isolation

DNA was isolated with the BioSprint 96 DNA Plant Kit (Qiagen, Hilden, Germany) in a BioSprint 96 workstation (Qiagen) following the manufacturer’s instructions. Leaf samples were ground using a Qiagen TissueLyser (Qiagen). Briefly, 20 mg of leaf tissue was placed into 2 mL tubes and 300 μL of RLT buffer (Qiagen) was added to each sample. One stainless steel 5 mm bead was used for each sample. Samples were then homogenized for 10 min at 30 Hz. Samples were centrifuged at 6000 g for 5 min and supernatant loaded into a 96-well plate with 200 μL isopropanol and 20 μL magnetic beads suspension (Qiagen). The beads were transferred consecutively into four other plates each with a premix, followed by a 4-min binding step and one bead collection step. The first plate was loaded with RPW buffer (guanidine thiocyanate buffer under patent protection). The second and third plates were loaded with 500 μL 96% ethanol. The fourth plate was loaded with 500 μL of 0.02% (v/v) using polyoxyethylene sorbitol anhydride monolaurate (Tween 20). DNA was eluted with 200 μL sterile water After isolation, DNA was assayed for concentration and purity by microfluidic gel electrophoresis with the Agilent 2200 TapeStation system (Agilent Technologies, Santa Clara, CA, USA). The average DNA yield was 50 ng μL^−1^ with an average 260:280 ratio of 1.85.

### 2.3. Library Preparation and Sequencing

Sequences of the known RGAs were obtained from Hunger et al. [22]. The Ion AmpliSeq Designer system (www.ampliseq.com) was used to generate target amplicons (about 200 bp in length) covering the RGAs sequences. The multiplexed amplicons were then used to generate barcoded libraries using the Ion AmpliSeq Library Kit 2.0 and the Ion Xpress barcoded adapters (Life Technologies, Carlsbad, CA, USA) to allow for discrimination between samples within a NGS run. Amplified libraries were quantified following the manufacturer’s recommendations. Barcoded libraries were combined to a final concentration of 7 pM, to achieve optimal yield of clonal templated Ion Sphere Particles (ISPs) (Life Technologies), for emulsion PCR (emPCR) and further ISP enrichment following the manufacturer’s recommendations. Sequencing was performed on 318 chips run on the Ion Torrent PGM and analyzed with the Torrent Suite v4.0.2 Software (Life Technologies). Quantification of prepared libraries was conducted by quantitative PCR using the Ion Library Quantification Kit (Life Technologies). Samples were run on the Ion Torrent PGM System (Life Technologies) as described by the manufacturer. The sequences were mapped against the sugar beet reference genome (RefBeet v. 1.2) and checked for known and novel mutations using Torrent Suite Software. The mutation positions identified were used for primer design and SNP Ampliseq assays.

### 2.4. Data Analysis

Alignment of the sequences against the reference sugar beet genome and base calling were performed using the Torrent Suite software. The identification of variants was performed by the Ion Torrent Variant Caller plugin software. SNP or multiple-nucleotide polymorphism (MNP) frequencies were calculated by dividing the number of mutations by the length of each RGA gene in bp. To analyze and visualize the clustering and differentiation of wild and cultivated beets, a principal coordinates analysis (PCoA) was performed on the SNPs data. PCoA was based on genetic distances calculated from SNP markers. PCoA was performed using ad hoc scripts and the R package GenABEL, version 2.12.2 [23]. Four samples with no mutations inside RGAs were removed from PCoA analysis. The Friedman’s analysis of variance (non-parametric ANOVA version for repeated measures) and the Kendall coefficient of concordance were used to determine differences between classes of beet individuals with respect to RGA genes [24,25]. The sequence alignment of the gene *Bv8_184910_pkon* in wild and cultivated plants was carried out using Clustal W with default parameters [26]. The non-parametric tests were performed using the STATISTICA v.13 software [27].

## 3. Results

Ion AmpliSeq was used to identify SNP within the sequence of twenty-one RGAs in individual DNA samples from wild and cultivated beets. The number of SNPs detected in 21 RGAs for each of the 96 individual samples is reported in Table 1. The number of SNPs per sample ranged from 0 to 278. The pollinator R740 (Sample ID 33), which was resistant to infection with rhizomonia, showed the highest number of within-RGAs mutations (278 SNPs) whereas samples 61 and 28 (31 SNPs) showed the lowest (Table 1). A total of 821 polymorphisms (763 SNPs and 58 MNPs) were identified inside 21 RGA genes in 95% (91/96) of the beet samples (Table 2). No SNPs were found inside RGAs in four beet samples. Of the 56 polymorphisms found in the gene *Bv8_184910_pkon*, 55 were SNPs and only 1 was a MNP. *Bv3_063740_usyf* located inside the Bvchr3.sca010 scaffold had the second highest number of identified SNPs and MNPs (127 mutations). The gene *Bv3_063740_usyf* showed the highest mutation frequency (2.91% for SNPs and 0.28% for MNPs) followed by *Bv7_171340_mgxu* (1.58% for SNPs and 0.19% for MNPs). 

Samples 8, 40 and 71 had the highest number of unique mutations (each with three unique SNPs) (Table 3).

**Figure 1:**
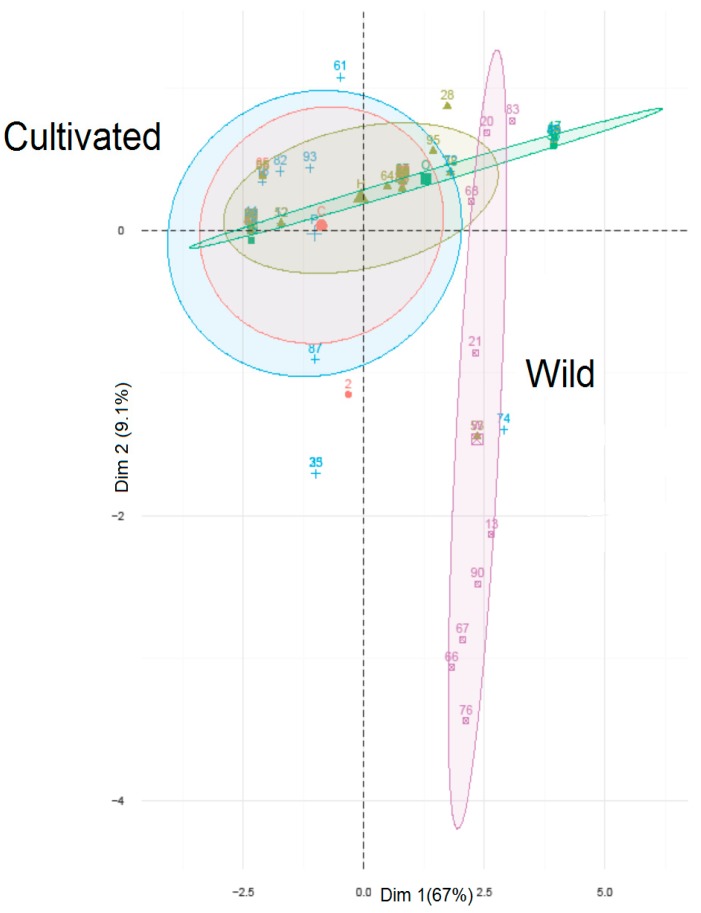
Principle coordinates analysis (PCoA) biplot of the gene *Bv8_184910_pkon* differentiates the wild and cultivated beets.

The results of Friedman’s Analysis of Variance (ANOVA) (*N* = 21, df = 4, *p*-value = 0.024) indicated that the mutations were distributed among five beet groups and the groups of beets were significantly different. The value of Kendall’s coefficient of concordance (0.13) suggests that the ranking (by number of polymorphisms) of RGA loci is not random with respect to beet groups. Table 3 shows unique MNP and Indel (insertion/deletion) detected in 21 genes among 96 individuals. The sample number 40 (R4430 carrying *Rz*2 gene for resistant to infection with rhizomonia) had the highest number of unique mutations (three unique SNPs). No mutation was identified in a number of RGA genes; ID 2, 3, 4, 13, 14, 16, 18, 19, 20 and 21 (Table 4). *Bv3_063740_usyf* which lies in the scaffold Bvchr3.sca010 on chromosome 3 had the highest number of SNPs within the wild beet group. The gene *Bv3_063740_usyf* showed the largest difference in SNP numbers between O-types and pollinators. Coefficient of variation (CV%) of the number of mutations between four types of beet plants varied from 0–60.0% (Table 4). The highest CV belonged to the gene *Bv7_172060_pnhe*. Among different beet groups, CMS lines (132) had the highest number of SNPs followed by wild beets (117) whereas the fewest SNPs (95) were found in the O-type class (Table 4). In PCoA, SNP data were used within-gene (i.e., RGA) to calculate genetic distances between samples: for all 21 RGA genes but one, it was not possible to clearly discriminate between wild and cultivated beets based on SNP genotypes. Only when the polymorphisms inside the *Bv8_184910_pkon* gene were used (including the CCCTCC sequence) to calculate genetic distances were wild and cultivated beets clearly differentiated. Figure 1 plots the first two dimensions from the PCoA of within-*Bv8_184910_pkon* SNP-based genetic distances. Sequence alignment of the *Bv8_184910_pkon* gene in wild and cultivated beets is reported in Figure S1.

## 4. Discussion

In the present study, we analyzed RGAs in the sugar beet genome to identify polymorphic sequences that differentiate wild accessions from domesticated beets. Although domestication is associated with the loss of resistance to biotic and abiotic stresses, it can be regarded as the selection of suitable wild accessions according to agriculturally relevant phenotypes [28]. The initial domestication event did sample the full range of natural variation in the wild progenitor populations and the characterization of such diversity at the functional locus level can result in more efficient preservation of plant genetic resources (PGR). More detailed information on the structure of genetic variation at the population level would allow a more efficient preservation of the genetic resources [29,30]. Differences in habitats may bring about different selective forces acting upon allele frequencies in populations. High throughput DNA sequencing is one of the approaches that have enabled the tracking of mutations that occurred during the domestication of crop plants. Different NGS platforms have been combined with bioinformatics tools to discover SNPs and mutations within genes [31]. Among the several available NGS platforms, the most popular are the Roche 454 Sequencer, the Illumina HiSeq and MiSeq and the Life Technologies Ion Torrent proton and personal genome machine (PGM) [32]. The Ion Torrent PGM used for polymorphism analysis herein is a high-throughput sequencer. The sequence is achieved by synthesis chemistry that uses a semiconductor-based, high-density array of micro-reaction chambers. The PGM’s sequence reads are about 100–200 bp and, thanks to a deep coverage, it is possible to detect mutations with low allele frequency.

In this study, we used the Ion Ampliseq approach to identify variants for 21 RGAs in 96 samples from five beet groups. This technology has been widely used in disease diagnosis in medical sciences [20], but our study is the first report of using Ion Ampliseq to screen 21 disease RGAs in beet germplasms. The high-quality AmpliSeq protocol was used in this study and the results showed that this technology was able to detect both single- and multi-nucleotide polymorphisms in domesticated and wild beets. The entire sequencing process, from library preparation to variant identification, was completed in two days. This re-sequencing approach will facilitate the rapid and cost-effective screening of RGA genes in beet germplasm. SNP flanking sequences from this study were uniquely mapped on the reference genome (RefBeet v. 1.2) and the AmpliSeq protocol on the Ion Proton platform showed robust sequencing results. Wild and cultivated accessions had 25 unique SNPs mapped onto all of the 21 RGAs. Four genes (*Bv5_094270_ijae Bv8_184910_pkon, Bv7_171340_mgxu,* and *Bv3_063740_usyf*) showed a density rate (variation at a specific position in the DNA sequence) of >1.0%. When the SNP data within the 21 RGAs were used to calculate distances between samples, no clear differentiation between wild and cultivated beets was observed from PCoA, save for one case. Only polymorphisms inside the RGA gene *Bv8_184910_pkon* could effectively differentiate wild from cultivated beet samples. These results suggest that most sources of resistance to rhizomania and nematodes in cultivated sugar beets have indeed been introgressed from *B. maritima* strains, hence they show no relevant genetic differences between the domesticated and wild germplasm. On the other hand, polymorphisms inside the *Bv8_184910_pkon* gene seem to be associated with a mutation which confers disease resistance. That mutation seems to have originated directly in cultivated beets in response to selective pressure related to rhizomania and nematode infections in field conditions. The *Bv8_184910_pkon* gene belongs to the receptor-like kinase (RLK) class of RGAs and its database annotation indicates that it relates to a hypothetical protein. A hypothetical protein is a protein whose existence has been predicted, but for which there is no experimental evidence that it is expressed in vivo. In silico methods can be used to predict hypothetical protein functions. The study of Beseli et al. [33] characterized *Cercospora nicotianae* hypothetical proteins in cercosporin resistance. Their results indicated that the *71cR* gene, encoding a hypothetical protein, was upregulated in *C. nicotianae* in response to cercosporin toxicity, and that the expression of this gene in the cercosporin-sensitive fungus *Neurospora crassa* can impart cercosporin resistance. RLKs are pattern recognition receptors (PRRs) that mediate pathogen-/microbe-associated molecular pattern (PAMP/MAMP) triggered immunity (PTI/MTI) to allow the recognition of a broad range of pathogens [34]. PAMP/MAMPs are conserved features of most pathogens, such as chitin, flagella, and lipopolysaccharides. *Xa21* in rice encodes an RLK involved in resistance to a bacterial disease caused by *Xanthomonas oryzae* (*Xoo*) [35]. In another study, Hunger et al. [22] found that some of the same RGAs used in the current study are involved in sugar beet’s resistance to infection with Cercospora. Genetic differentiation obtained using RGA-specific SNP markers may be useful for defining genetic diversity of a suite of random *R* genes in beet plants. They could also help to characterize the genetic structure and geographic distribution of the beet. In the present study, we identified a large number of allelic modifications in RGAs that may be related to important adaptive functions in sugar beet. These modifications appear to be potential targets for subsequent association studies aimed to identify SNP markers linked to disease resistance in sugar beet. SNP markers can be designed from RGAs around a target disease gene to construct an RGA genetic map for the specific target region. Such mapped, genome-wide RGAs and linked SNP markers are valuable tools to develop high-density *R*-gene genetic maps, target *R*-genes, co-localize QTLs, design diagnostic markers of *R*-genes for fine mapping, clone *R*-genes, and breed for resistance. 

In conclusion, the results of non-parametric tests confirmed that there is untapped variation in the wild materials. Further, they show that the sequence CCCTCC inside the gene *Bv8_184910_pkon*, responsible for differentiation of the wild from cultivated beets, can be used for rapid and convenient scanning of a large number of beet germplasms for identification of disease resistance in a time- and cost-effective assay. As most of the resistance genes share limited conserved domains, CCCTCC sequence information can be exploited to identify and clone unknown RGAs in wild beet plants. 

## Figures and Tables

**Table 1:** The number of single nucleotide polymorphisms (SNPs), in decreasing order, detected across the 21 resistance gene analogs (RGAs) in 96 beet samples.

Sample ID	Number of SNP	Genotype	Sample ID	Number of SNP	Genotype	Sample ID	Number of SNP	Genotype
33	278	P	83	151	W	5	120	O
82	248	P	9	150	O	53	118	H
67	225	W	25	150	P	17	116	O
90	222	W	46	149	H	16	114	P
96	218	H	76	149	W	58	113	O
88	206	P	55	148	H	50	112	O
73	190	C	62	148	H	56	112	H
80	189	P	92	148	H	70	110	H
2	188	C	75	147	H	79	109	P
42	187	C	22	145	H	86	103	H
63	184	H	14	144	H	91	103	P
8	180	H	77	144	P	45	98	O
23	180	H	49	143	C	65	96	C
74	176	P	6	142	P	39	92	H
59	173	H	3	141	P	78	90	H
7	171	H	41	139	O	26	86	P
1	168	O	48	139	P	37	86	H
15	168	H	18	138	O	12	85	P
66	168	W	27	137	O	93	84	P
71	166	P	31	136	H	10	82	P
32	165	H	34	135	P	36	81	H
40	163	P	94	134	P	19	75	O
24	160	H	30	132	H	21	71	W
52	160	H	47	132	H	38	71	H
72	157	P	89	132	P	87	71	P
13	155	W	11	129	P	35	57	O
43	155	H	64	129	H	60	57	P
20	154	W	95	129	H	28	31	H
4	152	P	51	127	C	61	31	P
54	152	H	81	126	C	-	-	-
68	152	W	57	121	C	-	-	-

C: cytoplasmic male sterile line; H: hybrid variety; O: O-type; P: pollinator; W: wild-type; ID: Identification.

**Table 2:** The number and frequency (%) of SNPs and multiple-nucleotide polymorphism (MNPs) detected across 21 RGAs in beet individuals.

Gene ID	NCBI Accession Number	Scaffold	Length (bp)	SNP (n)	MNP (n)	CDS	Non CDS	Freq. of SNP (%)	Freq. of MNP (%)
*Bv7_171470_ojty*	BU089572	Bvchr7.sca021	7827	57	2	31	18	0.73	0.03
*Bv2_043450_zhxk*	BU089578	Bvchr2.sca016	6687	0	0	0	0	0.00	0.00
*Bv9_225140_hpxq*	BH897910	Bvchr9.sca026	4738	0	0	0	0	0.00	0.00
*Bv6_147620_tzmc*	BU089568	Bvchr6.sca027	5232	0	0	0	0	0.00	0.00
*Bv6_152400_uaqg*	BU089560	Bvchr6.sca028	4164	9	1	0	10	0.22	0.02
*Bv7_172060_pnhe*	BU089566	Bvchr7.sca021	6568	29	1	12	18	0.44	0.02
*Bv8_184910_pkon*	BU089558	Bvchr8.sca001	4504	51	1	23	29	1.13	0.02
*Bv1_004790_mxoz*	BU089550	Bvchr1.sca001	5299	44	5	16	33	0.83	0.09
*Bv3_055670_fxjn*	BU089554	Bvchr3.sca003	6902	35	1	15	21	0.51	0.01
*Bv3_061550_ctrc*	BU089552	Bvchr3.sca008	4365	42	3	22	23	0.96	0.07
*Bv3_063740_usyf*	BH897908	Bvchr3.sca010	3986	116	11	61	66	2.91	0.28
*Bv5_094270_ijae*	BU089565	Bvchr5.sca002	5117	51	2	28	25	1.00	0.04
*Bv5_103960_ophq*	BU089548	Bvchr5.sca008	1783	0	0	0	0	0.00	0.00
*Bv6_148520_dzyz*	BU089547	Bvchr6.sca027	3628	0	0	0	0	0.00	0.00
*Bv7_157960_wiwi*	BU089579	Bvchr7.sca001	3891	14	1	15	0	0.36	0.03
*Bv7_160190_sdkg*	BU089562	Bvchr7.sca002	2767	0	0	0	0	0,00	0.00
*Bv7_171340_mgxu*	BU089575	Bvchr7.sca021	4685	74	9	44	39	1.58	0.19
*Bv7_175730_zcms*	BU089561	Bvchr7.sca021	3479	0	0	0	0	0.00	0.00
*Bv7u_181380_zcug*	BH897904	Bvchr7_un.sca003	3667	0	0	0	0	0.00	0.00
*Bv9_205550_geqf*	BH897907	Bvchr9.sca001	4424	0	0	0	0	0.00	0.00
*Bv9_218680_gqnp*	BU089549	Bvchr9.sca025	7235	0	0	0	0	0.00	0.00

bp: base pair; CDS: coding DNA sequence.

**Table 3:** List of unique MNPs and Indels (insertion/deletion) detected in 21 RGAs among 96 individuals.

Gene ID	Sample ID	MNP/Indel	RGA Class	Annotation
*Bv7_171470_ojty*	16	AG/GA	NLcc	Disease resistance protein At4g27190
	16	AGT/TTC		
	52	AGA/CGC		
	95	AA/CT		
	95	AG/GC		
*Bv8_184910_pkon*	13	CCCTCC/-	RLK	hypothetical protein
*Bv1_004790_mxoz*	8	GCCCC/CCCCT	Pto	U-box domain-containing protein 33 AltName: Full = Plant U-box protein 33; Includes: RecName: Full = E3 ubiquitin ligase; EC = 6.3.2.-; Includes: RecName: Full = Serine/threonine-protein kinase; EC = 2.7.11.-;
*Bv1_004790_mxoz*	71	-/AT	Pto	U-box domain-containing protein 33 AltName: Full = Plant U-box protein 33; Includes: RecName: Full = E3 ubiquitin ligase; EC = 6.3.2.-; Includes: RecName: Full = Serine/threonine-protein kinase; EC = 2.7.11.-;
*Bv3_055670_fxjn*	77	CA/-	Pto	U-box domain-containing protein 32 EC = 6.3.2.-; AltName: Full = Plant U-box protein 32;
*Bv3_061550_ctrc*	11	AT/GG	Pto	hypothetical protein
	31	GAC/GAC		
	35	CT/AC		
	54	TTG/TTG		
*Bv3_063740_usyf*	9	CG/GA	NLcc	Putative disease resistance protein RGA3 AltName: Full = RGA1-blb; AltName: Full = Blight resistance protein B149;
	9	GG/AC		
	38	CT/GC		
*Bv5_094270_ijae*	71	TA/AC	RLK	hypothetical protein
*Bv7_157960_wiwi Bv7_157960_wiwi Bv7_157960_wiwi*	41	TT/CC	CNL	Putative disease resistance protein RGA3 AltName: Full = RGA1-blb; AltName: Full = Blight resistance protein B149;
	57	CCA/CCA		
	67	GG/GG		
*Bv7_171340_mgxu*	12	AATAACCC/AATAACCC	Ncc	Probable disease resistance protein At4g27220
	40	AGT/CAC		
	40	AT/TC		
	40	ATG/CCA		
	68	-/TCCTTCA		

**Table 4:** The number of SNPs of RGAs per individual in five beet groups.

		Beet Groups						
Gene ID	Gene Name	C	H	O	P	W	Total	CV (%)
1	*Bv7_171470_ojty*	14	12	7	8	15	56	31.0
2	*Bv2_043450_zhxk*	0	0	0	0	0	0	0.0
3	*Bv9_225140_hpxq*	0	0	0	0	0	0	0.0
4	*Bv6_147620_tzmc*	0	0	0	0	0	0	0.0
5	*Bv6_152400_uaqg*	3	3	2	3	3	14	15.9
6	*Bv7_172060_pnhe*	3	1	3	1	1	9	60.0
7	*Bv8_184910_pkon*	12	11	6	11	9	49	24.3
8	*Bv1_004790_mxoz*	22	11	17	13	11	74	31.8
9	*Bv3_055670_fxjn*	4	3	4	2	3	16	26.1
10	*Bv3_061550_ctrc*	8	4	7	3	4	26	41.6
11	*Bv3_063740_usyf*	22	26	18	34	29	129	23.9
12	*Bv5_094270_ijae*	17	12	13	10	15	67	20.1
13	*Bv5_103960_ophq*	0	0	0	0	0	0	0.0
14	*Bv6_148520_dzyz*	0	0	0	0	0	0	0.0
15	*Bv7_157960_wiwi*	3	1	3	2	1	10	50.0
16	*Bv7_160190_sdkg*	0	0	0	0	0	0	0.0
17	*Bv7_171340_mgxu*	24	20	15	12	26	97	30.4
18	*Bv7_175730_zcms*	0	0	0	0	0	0	0.0
19	*Bv7u_181380_zcug*	0	0	0	0	0	0	0.0
20	*Bv9_205550_geqf*	0	0	0	0	0	0	0.0
21	*Bv9_218680_gqnp*	0	0	0	0	0	0	0.0
Total		132	104	95	99	117	621	

C: cytoplasmic male sterile line; CV: coefficient of variation; H: hybrid variety; O: O-type; P: pollinator; W: wild-type.

## Supplementary Materials

The following are available online at www.mdpi.com/2073-4425/8/10/264/s1. Figure S1: Sequence alignment of the *Bv8_184910_pkon* gene in wild and cultivated beets.

## References

[1] Food and Agriculture Organization of the United Nations (FAOSTAT) FAOSTAT Database. http://www.faostat.fao.org.

[2] Biancardi E., McGrath J.M., Panella L.W., Lewellen R.T., Stevanato P. (2010). Sugar beet. Tuber and Root Crops.

[3] Pavli O.I., Stevanato P., Biancardi E., Skaracis G.N. (2011). Achievements and prospects in breeding for rhizomania resistance in sugar beet. Field Crops Res..

[4] Stevanato P., Trebbi D., Panella L., Richardson K., Broccanello C., Pakish L., Fenwick A.L., Saccomani M. (2015). Identification and validation of a SNP marker linked to the gene *HsBvm-1* for nematode resistance in sugar beet. Plant Mol. Biol. Rep..

[5] Stevanato P., De Biaggi M., Skaracis G., Colombo M., Mandolino G., Biancardi E. (2001). The sea beet (*Beta vulgaris* L. ssp. *maritima*) of the Adriatic coast as source of resistance for sugar beet. Sugar Tech..

[6] Biancardi E., Lewellen R.T., De Biaggi M., Erichsen A.W., Stevanato P. (2002). The origin of rhizomania resistance in sugar beet. Euphytica.

[7] Sekhwal M.K., Li P., Lam I., Wang X., Cloutier S., Yo F.M. (2015). Disease resistance gene analogs (RGAs) in plants. Int. J. Mol. Sci..

[8] Liu J., Liu X., Dai L., Wang G. (2007). Recent progress in elucidating the structure, function and evolution of disease resistance genes in plants. J. Genet. Genom..

[9] Goodstein D.M., Shu S., Howson R., Neupane R., Hayes R.D., Fazo J., Mitros T., Dirks W., Hellsten U., Putnam N. (2012). Phytozome: A comparative platform for green plant genomics. Nucleic Acids Res..

[10] Monaco M.K., Stein J., Naithani S., Wei S., Dharmawardhana P., Kumari S., Amarasinghe V., Youens-Clark K., Thomason J., Preece J. (2014). Gramene 2013: Comparative plant genomics resources. Nucleic Acids Res..

[11] Nordberg H., Cantor M., Dusheyko S., Hua S., Poliakov A., Shabalov I., Smirnova T., Grigoriev I.V., Dubchak I. (2014). The genome portal of the department of energy joint genome institute: 2014 Updates. Nucleic Acids Res..

[12] Cai D., Kleine M., Kifle S., Harloff H.J., Sandal N.N., Marcker K.A., Klein-Lankhorst R.M., Salentijn M.J., Lange W., Stiekema W.J. (1997). Positional cloning of a gene for nematode resistance in sugar beet. Science.

[13] Dohm J.C., Minoche A.E., Holtgräwe D., Capella-Gutiérrez S., Zakrzewski F., Tafer H., Rupp O., Sörensen T.R., Stracke R., Reinhardt R. (2014). The genome of the recently domesticated crop plant sugar beet (*Beta vulgaris*). Nature.

[14] Sanger F., Nicklen S., Coulson A.R. (1977). DNA sequencing with chain-terminating inhibitors. Proc. Nat. Acad. Sci. USA.

[15] Liu L., Li Y., Li S., Hu N., He Y., Pong R., Lin D., Lu L., Law M. (2012). Comparison of Next-Generation sequencing systems. J. Biomed. Biotechnol..

[16] Stevanato P., Biscarini F. (2016). Digital PCR as new approach to SNP genotyping in sugar beet. Sugar Tech..

[17] Metzker M.L. (2010). Sequencing technologies—The next generation. Nat. Rev. Genet..

[18] Capistrano-Gossmann G.G., Ries D., Holtgrawe D., Minoche A., Kraft T., Frerichmann S.L.M., Soerensen T.R., Dohm J.C., Gonzalez I., Schilhable M. (2017). Crop wild relative populations of *Beta vulgaris* allow direct mapping of agronomically important genes. Nat. Commun..

[19] Grimmer M.K., Bean K.M.R., Luterbacher M.C., Stevens M., Asher M.J.C. (2008). Beet mild yellowing virus resistance derived from wild and cultivated *Beta* germplasm. Plant Breeding.

[20] Chang F., Li M.M. (2013). Clinical application of amplicon-based next-generation sequencing in cancer. Cancer Genet..

[21] Bai X., Zhang E., Ye H., Nandakumar V., Wang Z., Chen L., Tang C., Li J., Li H., Zhang W. (2014). PIK3CA and TP53 gene mutations in human breast cancer tumors frequently detected by Ion Torrent DNA sequencing. PLoS ONE.

[22] Hunger S., Di Gaspero G., Möhring S., Bellin D., Schäfer-Pregl R., Borchardt D.C., Durel C.E., Werber M., Weisshaar B., Salamini F. (2003). Isolation and linkage analysis of expressed disease-resistance gene analogues of sugar beet (*Beta vulgaris* L.). Genome.

[23] Aulchenko Y.S., Ripke S., Isaacs A., van Duijn C.M. (2007). GenABEL: An R library for genome-wide association analysis. Bioinformatics.

[24] Rousseeuw P.J., Ruts I., Tukey J.W. (1999). The Bagplot: A Bivariate Boxplot. Am. Stat..

[25] Bortz J., Lienert G., Boehnke K. (2000). Verteilungsfreie Methoden in der Biostatistik.

[26] Larkin M.A., Blackshields G., Brown N.P., Chenna R., McGettigan P.A., McWilliam H., Valentin F., Wallace I.M., Wilm A., Lopez R. (2007). Clustal W and Clustal X version 2.0. Bioinformatics.

[27] Statsoft STATISTICA. http://www.statsoft.com/Products/STATISTICA-Features/Version-12.

[28] Sahu K.K., Chattopadhyay D. (2017). Genome-wide sequence variations between wild and cultivated tomato species revisited by whole genome sequence mapping. BMC Genomics.

[29] Raamsdonk L.W.D. (1993). Wild and cultivated plants: The parallelism between evolution and domestication. Evol. Trends Pl..

[30] Letscher J.P.W., Frese L. (1993). Analysis of morphological variation in wild beet (*Beta vulgaris* L.) from Sicily. Genet. Resour. Crop Evol..

[31] Quail M.A., Smith M., Coupland P., Otto T.D., Harris S.R., Connor T.R., Bertoni A., Swerdlow H.P., Gu Y. (2012). A tale of three next generation sequencing platforms: Comparison of Ion Torrent, pacific biosciences and Illumnia MIseq sequencers. BMC Genomics.

[32] Rothberg J.M., Hinz W., Rearick T.M., Schultz J., Mileski W., Davey M., Leamon J.H., Johnson K., Milgrew M.J., Edwards M. (2011). An integrated semiconductor device enabling non-optical genome sequencing. Nature.

[33] Beseli A., Noar R., Daub M.E. (2015). Characterization of *Cercospora nicotianae* hypothetical proteins in Cercoporin resistance. PLoS ONE.

[34] Hammond-Kosack K.E., Jones J.D. (1997). Plant disease resistance genes. Annu. Rev. Plant Physiol. Plant Mol. Biol..

[35] Lee S.W., Han S.W., Sririyanum M., Park C.J., Seo Y.S., Ronald P.C. (2009). A type I-secreted, sulfated peptide triggers Xa21-mediated innate immunity. Science.

