# Supplementary material for: Targeted Next-Generation Sequencing Identification of Mutations in Disease Resistance Gene Analogs (RGAs) in Wild and Cultivated Beets
Source: Genes (Basel). 2017 Oct 11;8(10):264. doi: 10.3390/genes8100264 (PMC5664114; doi:10.3390/genes8100264)
Supplement: Supplementary file 1 [file genes-08-00264-s001.pdf]

```
                           ******************************************************************************************************************************************************
Bv8_184910_pkon_cultivated TTTCCTTTCTAATCATTTTCTTGTTTAACTTTCTATTATTATCAACAACAATTATGCTAATAGCAAAACATACTATAATTGTAGCAAAGAATTAACTAAACAGTATTCATAGGCAACTCACCAGGAGCAATGTAGCCACAAGAACCGGCG   150
Bv8_184910_pkon_wild       TTTCCTTTCTAATCATTTTCTTGTTTAACTTTCTATTATTATCAACAACAATTATGCTAATAGCAAAACATACTATAATTGTAGCAAAGAATTAACTAAACAGTATTCATAGGCAACTCACCAGGAGCAATGTAGCCACAAGAACCGGCG   150

                           *** ** * ***  *********** *********************************************** ****************************************************************************
Bv8_184910_pkon_cultivated ATGCCCAAAATCATTTTAGTCCCTTTTCCGATAGCGTCCACTACTTTCGCCACTCCGAAATCCGCCACCTTAGAACCGTAATCACCATCCAAAAGGATGTTATTTGATTTCACATCTCTATGAACAATAGCCGGGACACAATCATGGTGC   300
Bv8_184910_pkon_wild       ATGTCCTACATCCCTTTAGTCCCTTCTCCGATAGCGTCCACTACTTTCGCCACTCCGAAATCCGCCACCTTAGCACCGTAATCACCATCCAAAAGGATGTTATTTGATTTCACATCTCTATGAACAATAGCCGGGACACAATCATGGTGC   300

                           ******************************************************* ******      ************************  *** ************************** *************************
Bv8_184910_pkon_cultivated AAGTACGAAAGACCTTCAGCAGCATCGAGGGCGATTCGATACCTCGTAGGCCAATTTAATAA------TTTAGAACTATGCAACAAATCACCAAAACAACCATTTGGCATATACTCATAAACAACCAACTTACAATCTTTAGTCTTGCAA   450
Bv8_184910_pkon_wild       AAGTACGAAAGACCTTCAGCAGCATCGAGGGCGATTCGATACCTCGTAGGCCAATCTAATAACCCTCCTTTAGAACTATGCAACAAATCACCCGAACGACCATTTGGCATATACTCATAAACAATCAACTTACAATCTTTAGTCTTGCAA   450

                           ******* ** ** ***************************** ************** ******** ********* ** * ** *********************************** ***** ****** ***************
Bv8_184910_pkon_cultivated CAACACCTTATTCTTACTATATTCTTATGCCTAATCTTCCCCACCGTTTCGACTTCAGTTTCGAATCTATCATCGTCAATACTTCAACCATTTTCGACATCTCCTCCGTCTCCATCGACAATTTTATTCGAAAAACCCCATAGTTTCTTC   600
Bv8_184910_pkon_wild       CAACACCCTACTCCTACTATATTCTTATGCCTAATCTTCCCCATCGTTTCGACTTCAGCTTCGAATCGATCATCGTCGATGCCTCGACCATTTTCGACATCTCCTCCGTCTCCATCGACAACTTTATCCGAAAACCCCCATAGTTTCTTC   600

                           ************************************ ***** **************************************** ************* ************************** *********************** *
Bv8_184910_pkon_cultivated ACTGCTACAGCCTCACCATTGCTAAGAACAGCTTTGCACACTCTCCCTGAAGCTCCTGTTCCTATCACATTATCTTCATCTAACCAATCCAAAATCTAATATTCACTAAAACCCAATTTGTGAACCGACATTATAGTCCATTTTGATTAA   750
Bv8_184910_pkon_wild       ACTGCTACAGCCTCACCATTGCTAAGAACAGCTTTGGACACTATCCCTGAAGCTCCTGTTCCTATCACATTATCTTCATCTAAACAATCCAAAATCTGATATTCACTAAAACCCAATTTGTGAATCGACATTATAGTCCATTTTGATTGA   750

                           **** ******************************************** ******************************************************** ******************** **********************
Bv8_184910_pkon_cultivated TCAACCGTTTGTTTCACTTGGTTGAAATTCTTGTACTTGTAATAAAACCCCACTACACCCACTAAGAAAATAACCGCAGCAAGAACAAATATTGTTCTTAACAACCTTAAATACCTCTGTTTTTTCACCTCAATTCTACTATCACATAAA   900
Bv8_184910_pkon_wild       TCAATCGTTTGTTTCACTTGGTTGAAATTCTTGTACTTGTAATAAAACCTCACTACACCCACTAAGAAAATAACCGCAGCAAGAACAAATATTGTTCTTAACAACCCTAAATACCTCTGTTTTTTCATCTCAATTCTACTATCACATAAA   900

                           ********** * *************************************** ***************** ************************************ ******************************************
Bv8_184910_pkon_cultivated CCAGATATCTAACCACATAATCCAGGGTTCCCCATAAATGAACTCTTATACATTTCCTTAGCAAACAATGCCGGAAGTTCGCCGGATAATCGATTATTCGACAAATTAAACTGATTCAACTTCAAGTTCTGCAATTCATCTGGAACTGGA  1050
Bv8_184910_pkon_wild       CCAGATATCTCAGCACATAATCCAGGGTTCCCCATAAATGAACTCTTATACACTTCCTTAGCAAACAATGACGGAAGTTCGCCGGATAATCGATTATTCGACAAATTGAACTGATTCAACTTCAAGTTCTGCAATTCATCTGGAACTGGA  1050

                           ****** ***********************************    ** *  * ************************************************************************************************
Bv8_184910_pkon_cultivated CCAATTAACTGATTATTCGACAAATCGAGGTAATTAAGCGTTATAAACTTCCCAATTTCCGCCGGAATTCTCCCGGAAAAACCATTACTTCCTAAATTCAAATCATTAAGTTTCTTCATTTCCGTAATCTCAGATGAAATCTCACCACTT  1200
Bv8_184910_pkon_wild       CCAATTGACTGATTATTCGACAAATCGAGGTAATTAAGCGTTGGCTACATTTCTATTTCCGCCGGAATTCTCCCGGAAAAACCATTACTTCCTAAATTCAAATCATTAAGTTTCTTCATTTCCGTAATCTCAGATGAAATCTCACCACTT  1200

                           ******************************************************************************************************************************************************
Bv8_184910_pkon_cultivated AACTCATTATTCTCCAAATTTAACCTCCCTAACTCCCTCAAATTCACCATAGAATCCGGCAATGACCCAGAAAACTGATTATCATTCGCAACAAAACTCAATAAATTGCTTAAAAACCCAATCTCCTCCGGAATTTTCCCAGAAAAATTA  1350
Bv8_184910_pkon_wild       AACTCATTATTCTCCAAATTTAACCTCCCTAACTCCCTCAAATTCACCATAGAATCCGGCAATGACCCAGAAAACTGATTATCATTCGCAACAAAACTCAATAAATTGCTTAAAAACCCAATCTCCTCCGGAATTTTCCCAGAAAAATTA  1350

                           ******************************************************************************************************************************************************
Bv8_184910_pkon_cultivated TTATTCGAAATCAAAAGCTGCGATAAATTCAAACCCGAAGCAATTGATTTCGATATTTCCCCAGAAAACAAATTATACGAAAGTTCAAGCAATGATAAATGAGGTAACCCCCAGAAACTCGGTGGTACATTGCCGGAAAATCGATTATTA  1500
Bv8_184910_pkon_wild       TTATTCGAAATCAAAAGCTGCGATAAATTCAAACCCGAAGCAATTGATTTCGATATTTCCCCAGAAAACAAATTATACGAAAGTTCAAGCAATGATAAATGAGGTAACCCCCAGAAACTCGGTGGTACATTGCCGGAAAATCGATTATTA  1500

                           ******************************************************************************************************************************************************
Bv8_184910_pkon_cultivated CTCAACCTTACTCTCGTTAAACTCCTACACTCTGATAAACTCGCCGGAATTTCGCCGGAAAACGAGTTATCAATCATCAACAACTGAGTTAACTCACCTTTAGAACACAACCCAGGTGGAATCTCCCCACTAAAATTATTATTCGACACA  1650
Bv8_184910_pkon_wild       CTCAACCTTACTCTCGTTAAACTCCTACACTCTGATAAACTCGCCGGAATTTCGCCGGAAAACGAGTTATCAATCATCAACAACTGAGTTAACTCACCTTTAGAACACAACCCAGGTGGAATCTCCCCACTAAAATTATTATTCGACACA  1650

                           ******************************************************************************************************************************************************
Bv8_184910_pkon_cultivated TCAATAAGCTCCAACCTCGAATTCTCCCCGAGTTTCTTCGGTAACTCACCAGAAAGTTTATTACTAAAAAGCTTCAACTCGTATAAATTCACCGAGTTCGCTATACTCTCAGGAACATCACCCACCAACTCATTCTCGTACAGATTCAAC  1800
Bv8_184910_pkon_wild       TCAATAAGCTCCAACCTCGAATTCTCCCCGAGTTTCTTCGGTAACTCACCAGAAAGTTTATTACTAAAAAGCTTCAACTCGTATAAATTCACCGAGTTCGCTATACTCTCAGGAACATCACCCACCAACTCATTCTCGTACAGATTCAAC  1800

                           ******************************************************************************************************************************************************
Bv8_184910_pkon_cultivated GACGCGAGTTGCAACTGAGTCAAATCGTCGGGAATCGTCCCGTTGAGCTTGTTCATCGCAGCATCAAACATCCTCAACGACCTCATCTTCAACCACCCAGAAGACGGCAACTCGCCGCTGAACTCGTTCCCGTAAAGCTCAATCTGCACC  1950
Bv8_184910_pkon_wild       GACGCGAGTTGCAACTGAGTCAAATCGTCGGGAATCGTCCCGTTGAGCTTGTTCATCGCAGCATCAAACATCCTCAACGACCTCATCTTCAACCACCCAGAAGACGGCAACTCGCCGCTGAACTCGTTCCCGTAAAGCTCAATCTGCACC  1950

                           ******************************************************************************************************************************************************
Bv8_184910_pkon_cultivated GCACTCGTCAACTCAGTGATCGAACTCGGTATCCTCCCGCTCAAACCGTTGCTCGACACGTCGAAGTTGATCACCTTCCCGAGTCGACTCACCGAGTCAGGCAACTCGCCGAACAAGCTGCAGTTAGCAACCCAGAAATACTCGAGGCTC  2100
Bv8_184910_pkon_wild       GCACTCGTCAACTCAGTGATCGAACTCGGTATCCTCCCGCTCAAACCGTTGCTCGACACGTCGAAGTTGATCACCTTCCCGAGTCGACTCACCGAGTCAGGCAACTCGCCGAACAAGCTGCAGTTAGCAACCCAGAAATACTCGAGGCTC  2100

                           ******************************************************************************************************************************************************
Bv8_184910_pkon_cultivated GCTAAGTTACCGATCTCAGGACCGATTTGACTCGGCCTAAACGGGTTGTAACTCAGATTTAAGGTCTTCAGAGTAGTAACATTGGTCAAGAACATGGGGAAATCTCCGTCGAGTAGGTTATCGACCAATGAAATCTCCTCAAGGTTCCGG  2250
Bv8_184910_pkon_wild       GCTAAGTTACCGATCTCAGGACCGATTTGACTCGGCCTAAACGGGTTGTAACTCAGATTTAAGGTCTTCAGAGTAGTAACATTGGTCAAGAACATGGGGAAATCTCCGTCGAGTAGGTTATCGACCAATGAAATCTCCTCAAGGTTCCGG  2250

                           ******************************************************************************************************************************************************
Bv8_184910_pkon_cultivated AAAGTTCCGAAACTCGCCGGAACATCGCCGGAGAAGTTATTCCCGCTCAAATCCAAGTACCGGAGACTGGTTATTGATGCAAGAGAACCAGGTATTTCACCCACGAGTGTATTAAGCGAAAGGTCGAGATAAACGAGATTACTACAGTTG  2400
Bv8_184910_pkon_wild       AAAGTTCCGAAACTCGCCGGAACATCGCCGGAGAAGTTATTCCCGCTCAAATCCAAGTACCGGAGACTGGTTATTGATGCAAGAGAACCAGGTATTTCACCCACGAGTGTATTAAGCGAAAGGTCGAGATAAACGAGATTACTACAGTTG  2400
```

```
                           ******************************************************************************************************************************************************
Bv8_184910_pkon_cultivated GAGATATTAGAAGGTAAAGATGAGTTGATGAAGTTGTTAGAGAATGAAAGAGATGAGAGAGAATGTAGACGACATAAGATAAGAGGAAATGGTCCAACAAGATTGAAAGAAGTGAAGTTAAGAGAGATGATTGAGGAAGTAGTAGAGTCG  2550
Bv8_184910_pkon_wild       GAGATATTAGAAGGTAAAGATGAGTTGATGAAGTTGTTAGAGAATGAAAGAGATGAGAGAGAATGTAGACGACATAAGATAAGAGGAAATGGTCCAACAAGATTGAAAGAAGTGAAGTTAAGAGAGATGATTGAGGAAGTAGTAGAGTCG  2550

                           ******************************************************************************************************************************************************
Bv8_184910_pkon_cultivated CAAGTGACTGCAGACCAATTGCATGGTGTGTCATGGCGAGTGTTCCAGTTAGAGAGAGAGTTTGTTGGGTCGTCTAAAGTGAGTTTGAGGTGTTGAAGGTAAAGACCTTCTTGGTTTAGAGAGAGTGTTTGTGGGAAAGAGATGAAGATG  2700
Bv8_184910_pkon_wild       CAAGTGACTGCAGACCAATTGCATGGTGTGTCATGGCGAGTGTTCCAGTTAGAGAGAGAGTTTGTTGGGTCGTCTAAAGTGAGTTTGAGGTGTTGAAGGTAAAGACCTTCTTGGTTTAGAGAGAGTGTTTGTGGGAAAGAGATGAAGATG  2700

                           ******************************************************************************************************************************************************
Bv8_184910_pkon_cultivated GAGATGATGAAGATGGTGATGAGAGTGAGAGGGAACATTTTAGTGGAGGAGAGAGAGAAAGCATGGAATGGAAGAAGGAAGAATGGGAGGGGAGAAAGTGTGAAGAAGAGTGAGTGGGTGGTTTTTTTTTTTGTGTTGTGGTGTTTGGGA  2850
Bv8_184910_pkon_wild       GAGATGATGAAGATGGTGATGAGAGTGAGAGGGAACATTTTAGTGGAGGAGAGAGAGAAAGCATGGAATGGAAGAAGGAAGAATGGGAGGGGAGAAAGTGTGAAGAAGAGTGAGTGGGTGGTTTTTTTTTTTGTGTTGTGGTGTTTGGGA  2850

                           ******************************************************************************************************************************************************
Bv8_184910_pkon_cultivated GTGAAATTTGAACGTGGTGATATAGGGAGTTAAGTTAGTATTAAAGTGGTATAGGATGAGGATTGAGGGGTGAGAGAGGGGGGAGTGTAATTATTATGGTCCGTTCGGTATTAGTGTATCTAATTTAATTCAGTGTAAAAGAATATATCA  3000
Bv8_184910_pkon_wild       GTGAAATTTGAACGTGGTGATATAGGGAGTTAAGTTAGTATTAAAGTGGTATAGGATGAGGATTGAGGGGTGAGAGAGGGGGGAGTGTAATTATTATGGTCCGTTCGGTATTAGTGTATCTAATTTAATTCAGTGTAAAAGAATATATCA  3000

                           ******************************************************************************************************************************************************
Bv8_184910_pkon_cultivated GTTTTGAATTTTGGTTTTAGGAATACAAAAGTAATACTCCCTCCGTTTCAAAATATAGTTTTCGTGTTCTTTTGTATAGTTTTCAAGACGTATATTTGACTTATTATATCTTCTATTTTGTATTTGTAAAAAATATAAAAAATCGATATT  3150
Bv8_184910_pkon_wild       GTTTTGAATTTTGGTTTTAGGAATACAAAAGTAATACTCCCTCCGTTTCAAAATATAGTTTTCGTGTTCTTTTGTATAGTTTTCAAGACGTATATTTGACTTATTATATCTTCTATTTTGTATTTGTAAAAAATATAAAAAATCGATATT  3150

                           ******************************************************************************************************************************************************
Bv8_184910_pkon_cultivated ATGAATGTACATATTGAGACATAACAAGATCTGACATGAATATGTTTTTTTTGTATACATTTAATTAAATATTTGTTGAAATAGTAATTATATTTGAGACGGATGAATTATGATATAAAGGATATAATATATTTAAGCTTAATCATATTT  3300
Bv8_184910_pkon_wild       ATGAATGTACATATTGAGACATAACAAGATCTGACATGAATATGTTTTTTTTGTATACATTTAATTAAATATTTGTTGAAATAGTAATTATATTTGAGACGGATGAATTATGATATAAAGGATATAATATATTTAAGCTTAATCATATTT  3300

                           ******************************************************************************************************************************************************
Bv8_184910_pkon_cultivated ATATTATACATGTCAATTTTTAAACTGATGACAAGAGTATACTGAAATTACTTTTTTTGTTTTTTGGTTTTCGGAAAAAGTGAAAACTTAAAACGATAATGAAGAATCCTTATATTTCCTTTTTAATTACTATATTTTTGTCGTTAATAT  3450
Bv8_184910_pkon_wild       ATATTATACATGTCAATTTTTAAACTGATGACAAGAGTATACTGAAATTACTTTTTTTGTTTTTTGGTTTTCGGAAAAAGTGAAAACTTAAAACGATAATGAAGAATCCTTATATTTCCTTTTTAATTACTATATTTTTGTCGTTAATAT  3450

                           ******************************************************************************************************************************************************
Bv8_184910_pkon_cultivated GAAACAATTAAGGAAAGGGGAATGATATTTAACAATAAAAAGCTACAAACTAAAATGGAGGATATTTAACAATAAATAAACATTGATGGATAGTTATAACTTTTTTATTGATTATCTTATTATCTAATTAATCCTACAATGTGTTAAAAA  3600
Bv8_184910_pkon_wild       GAAACAATTAAGGAAAGGGGAATGATATTTAACAATAAAAAGCTACAAACTAAAATGGAGGATATTTAACAATAAATAAACATTGATGGATAGTTATAACTTTTTTATTGATTATCTTATTATCTAATTAATCCTACAATGTGTTAAAAA  3600

                           ******************************************************************************************************************************************************
Bv8_184910_pkon_cultivated ATATGTTTTCCAACCTAGATTTATTAAGATTCGTTGTATAGTTGTCTTGAAACTTTGAATGAATGTATGCCTTGTAGGACATTGATTAAATGCTTGTATTTGGAATATAGACTTTGGTTAACTATTGATGCTCTTTATTAAATAGCTACA  3750
Bv8_184910_pkon_wild       ATATGTTTTCCAACCTAGATTTATTAAGATTCGTTGTATAGTTGTCTTGAAACTTTGAATGAATGTATGCCTTGTAGGACATTGATTAAATGCTTGTATTTGGAATATAGACTTTGGTTAACTATTGATGCTCTTTATTAAATAGCTACA  3750

                           ******************************************************************************************************************************************************
Bv8_184910_pkon_cultivated TTGAAAATTTAGAGTTTTATTTACATTTCCATATATGTTATTAAGAAGAACCGAGCATGACTTGTAGTACCTAGTAATGCTACAATAGTGAAAAAAAATATTATTTCATGTGAGTCCTTGATCAAATTTTTTGTTTTAAAAAGTGAGTAN  3900
Bv8_184910_pkon_wild       TTGAAAATTTAGAGTTTTATTTACATTTCCATATATGTTATTAAGAAGAACCGAGCATGACTTGTAGTACCTAGTAATGCTACAATAGTGAAAAAAAATATTATTTCATGTGAGTCCTTGATCAAATTTTTTGTTTTAAAAAGTGAGTAN  3900

                           ******************************************************************************************************************************************************
Bv8_184910_pkon_cultivated NNNNNNNNNNNNNNNNNNNNNNNNNNNNNNNNNNNNNNNNNNNNNNNNNNNNNNNNNNNNNNNNNNNNNNNNNNNNNNNNNNNNNNNNNNNNNNNNNNNNNNNNNNNNNNNNNNATAGGAAATTGCATTTCCTAGGAAATTACTCACCAA  4050
Bv8_184910_pkon_wild       NNNNNNNNNNNNNNNNNNNNNNNNNNNNNNNNNNNNNNNNNNNNNNNNNNNNNNNNNNNNNNNNNNNNNNNNNNNNNNNNNNNNNNNNNNNNNNNNNNNNNNNNNNNNNNNNNNATAGGAAATTGCATTTCCTAGGAAATTACTCACCAA  4050

                           ******************************************************************************************************************************************************
Bv8_184910_pkon_cultivated GGGGGGGCTTGGTGAGTTATTTCCCATGTTTCATGGGAAATTCTATTTCCCTTGGAATTTGTATTTCCTATGCTTCTCCCATTTCCCTCCAACCAAACACAACATCCATAATGTATTTCCTAGGAATTTGTATTTCCAAGGAAAATAGCT  4200
Bv8_184910_pkon_wild       GGGGGGGCTTGGTGAGTTATTTCCCATGTTTCATGGGAAATTCTATTTCCCTTGGAATTTGTATTTCCTATGCTTCTCCCATTTCCCTCCAACCAAACACAACATCCATAATGTATTTCCTAGGAATTTGTATTTCCAAGGAAAATAGCT  4200

                           ******************************************************************************************************************************************************
Bv8_184910_pkon_cultivated TCCAACCAAACAACACCTAAGATGACAATGAGAATATGACTTTCAAATATGGGATATTGAGGAAGATTAATTAGGTGTTTGTATGAGAGGTGTTTAGAAAATTATATGTAGGACACATGCTATATATACCTAAAATAGGTTGTAATAATG  4350
Bv8_184910_pkon_wild       TCCAACCAAACAACACCTAAGATGACAATGAGAATATGACTTTCAAATATGGGATATTGAGGAAGATTAATTAGGTGTTTGTATGAGAGGTGTTTAGAAAATTATATGTAGGACACATGCTATATATACCTAAAATAGGTTGTAATAATG  4350

                           ******************************************************************************************************************************************************
Bv8_184910_pkon_cultivated TGTGAGTTGTATTGGAAATTTGGAATTATCCATATATACCTTATAGATAAGGGCTTTTCTGTGTCGGGTGTGTTTTCGTTACTTTTTTTTACATAAAAACTTCCTTTTCTAAGAAGGATTACCTTATTTAAATCCGAATTTGAGGCTCTC  4500
Bv8_184910_pkon_wild       TGTGAGTTGTATTGGAAATTTGGAATTATCCATATATACCTTATAGATAAGGGCTTTTCTGTGTCGGGTGTGTTTTCGTTACTTTTTTTTACATAAAAACTTCCTTTTCTAAGAAGGATTACCTTATTTAAATCCGAATTTGAGGCTCTC  4500

                           *****
Bv8_184910_pkon_cultivated TTCTA  4505
Bv8_184910_pkon_wild       TTCTA  4505
```
